# The Occurrence of Folate Biosynthesis Genes in Lactic Acid Bacteria from Different Sources

**DOI:** 10.17113/ftb.61.02.23.7929

**Published:** 2023-06

**Authors:** Fenny Amilia Mahara, Lilis Nuraida, Hanifah Nuryani Lioe, Siti Nurjanah

**Affiliations:** 1 Department of Food Science and Technology, Faculty of Agricultural Engineering, and Technology, IPB University (Bogor Agricultural University), 16680 Bogor, Indonesia; 2 Southeast Asian Food and Agricultural Science and Technology (SEAFAST) Center, IPB University (Bogor Agricultural University), 16680 Bogor, Indonesia

**Keywords:** extracellular folate producer, folate biosynthetic genes, lactic acid bacteria

## Abstract

**Research background:**

Lactic acid bacteria (LAB) are known to produce folate. However, this ability is highly strain-dependent. Folate synthesis in specific LAB strains is affected by the availability of folate, which can be consumed by other LAB under certain conditions. Moreover, differences in folate synthesis capabilities are related to the presence of folate biosynthesis-related genes and regulation of this pathway.

**Experimental approach:**

As basic information to better understand the regulation of folate biosynthesis among different LAB species and strains, folate biosynthetic genes were screened and identified in folate-producing and non-folate-producing LAB isolated from various local food sources in Indonesia. The extracellular folate productivity amounts of the isolates were analyzed using high-performance liquid chromatography with a diode array detector (HPLC-DAD).

**Results and conclusions:**

Eleven of the thirteen tested LAB isolates had all of the eight genes involved in folate biosynthesis (*folE*, *folQ*, *folB*, *folK*, *folP*, *folC_1_*, *folA* and *folC_2_*). Furthermore, these isolates produced extracellular folate ranging from 10.37 to 31.10 µg/mL. In contrast, two non-folate-producing isolates lacked several folate biosynthetic genes, such as *folQ*, *folP* and *folA*, which is possibly the reason for their inability to synthesize folate *de novo*. Phylogenetic tree construction revealed that the folate biosynthetic genes (excluding *folK* and *folP*) from six distinct species of folate-producing LAB isolates were monophyletic with homologous genes from other LAB species in the database.

**Novelty and scientific contribution:**

In this study, the distribution of folate biosynthetic genes in various LAB species was determined. The findings from this research support the use of folate biosynthesis marker genes in the genotypic screening for folate-producing LAB.

## INTRODUCTION

Folate is a micronutrient required for growth, particularly during foetal development. Deficiency in this vitamin can lead to various disorders, such as megaloblastic anaemia, neural tube defects, coronary heart disease, and cancer risk (*1*). Thus, folate supplementation is one of the world's primary nutritional goals, especially in pregnant women (*2*, *3*).

Recently, folate-producing microorganisms have been increasingly used to produce natural folate-rich food products (*4*). In addition to a variety of green plants (*5*), folate can be synthesized by certain microbes, such as lactic acid bacteria (LAB) (*6*–*9*). Microorganism-produced folate is more available for absorption by the human body and, hence, more effective in providing folate needs (*10*, *11*).

Nevertheless, the capacity of LAB to synthesize folate is highly strain-dependent (*7*, *9*, *12*). Various attempts have been made to select superior folate producers by exploiting diverse food sources (*7*, *8*, *13*–*15*). Growth optimization is also frequently implemented to boost folate production (*11*, *16*, *17*). However, the presence of folate in the media can affect the bacterial ability to produce it, with some folate-producing strains consuming the available folate in the media rather than resynthesizing it (*18*). This might be due to the efficient metabolic regulation in microbes (*19*), and possibly the presence of feedback inhibition mechanisms in the regulation of folate biosynthesis (*20*, *21*). Consequently, folate in the media may inhibit or inactivate several enzymes involved in folate biosynthesis (*18*).

Folate biosynthesis requires three main building blocks, namely: (*i*) the pteridine moiety (6-hydroxymethyl-7,8-dihydropterin pyrophosphate (DHPPP)), (*ii*) 4-aminobenzoic acid (*p*-aminobenzoic acid or PABA), and (*iii*) glutamate. Most LAB cannot synthesize PABA and glutamate (*12*, *22*), which need to be supplied in the medium. Hence, the folate biosynthetic pathway can be divided into two phases, *i.e*. the formation of the pteridine moiety (DHPPP) and the combination of the three constituents of folate.

Eight folate biosynthetic enzymes are involved in the conversion of the guanosine triphosphate (GTP) precursor into tetrahydrofolate (THF) polyglutamate. Initially, GTP cyclohydrolase I (encoded by the *folE* gene) catalyzes the conversion of GTP to 7,8-dihydroneopterin triphosphate. Enzyme dITP/XTP pyrophosphatase (encoded by the *folQ* gene) hydrolyzes dihydroneopterin triphosphate to dihydroneopterin monophosphate, which is then hydrolyzed further to dihydroneopterin by a specific phosphatase. The biosynthetic pathway continues with the conversion of dihydroneopterin to 6-hydroxymethyl-7,8-dihydropterin by dihydroneopterin aldolase, encoded by the *folB* gene, and further converted to DHPPP by hydroxymethyl dihydropterin pyrophosphokinase (HPPK), encoded by *folK*. DHPPP and PABA are transformed into dihydropteroate by dihydropteroate synthase (DHPS), encoded by *folP*, and then conjugated with glutamate to become dihydrofolate by dihydrofolate synthase (encoded by *folC_1_*). Dihydrofolate is an inactive form of folate; thus, it must be reduced by dihydrofolate reductase (encoded by *folA*) into the active form, tetrahydrofolate (THF). The newly synthesized THF is in the monoglutamate form, hence, requiring the activity of folylpolyglutamate synthase (encoded by *folC_2_*) to add multiple glutamate residues to form THF polyglutamate.

*In silico* research by de Crécy-Lagard *et al.* (*23*) showed that two folate biosynthetic genes, *folK* (encoding HPPK enzyme) and *folP* (encoding DHPS enzyme), can be used as signature genes of folate biosynthesis. The proteins encoded by these two genes were found in all folate-producing bacteria in an investigation of nearly 400 bacterial genome sequences. Turpin *et al*. (*24*) also performed molecular screening of the two signature genes on 152 strains of six distinct LAB species. They found that 98 % of the isolates (150 strains) possessed these two genes, implying that these isolates could produce folate. However, Greppi *et al*. (*9*) discovered that despite having both hallmark genes, 56 of these isolates could not synthesize folate and instead consumed folate in the medium. Therefore, the detection of *folK* and *folP* genes is insufficient to determine folate production capability in LAB.

This research aims to determine the occurrence of genes encoding folate biosynthetic enzymes in various folate-producing and folate-consuming LAB species isolated from diverse local food sources in Indonesia. Results of the distribution of folate biosynthetic genes in distinct LAB species in this study can be useful in understanding variations in the regulation of folate biosynthesis across different LAB species and strains.

## MATERIALS AND METHODS

### LAB isolates and growth conditions

The LAB isolates used in this study (Table 1 (*18*, *25*)) were taken from the culture collections of the SEAFAST Center, IPB University, Bogor, Indonesia. These isolates exhibited varying growth abilities in folate-free media (*18*). *Lactiplantibacillus plantarum* WCFS1 was used as a positive control for folate gene detection because all folate biosynthesis genes are present in its genome (*24*–*26*). All LAB isolates were stored in a mixture of de Man, Rogosa and Sharpe broth (MRSB; CM0359, Oxoid Ltd., Basingstoke, UK) and 20 % glycerol at −20 °C and revived in MRSB before use.

**Table 1:** Lactic acid bacteria used in this study

No	Isolate	Source	GenBank accession no.	Reference
1	*Lactiplantibacillus plantarum* WCFS1	Human saliva	NC_004567.2	(*25*)
2	*Lactiplantibacillus plantarum* 4C261	Salted mustard	OM980095	(*18*)
3	*Lactiplantibacillus plantarum* R12	Breast milk	MG952229	(*18*)
4	*Limosilactobacillus fermentum* JK13	Kefir granules	ON005305.1	(*18*)
5	*Limosilactobacillus fermentum* JK16	Kefir granules	ON025957.1	(*18*)
6	*Limosilactobacillus fermentum* BK27	Sticky rice tapai	MG934339	(*18*)
7	*Limosilactobacillus fermentum* BG7	Kefir granules	ON005183	(*18*)
8	*Lacticaseibacillus rhamnosus* R23	Breast milk	MF689061	(*18*)
9	*Lacticaseibacillus rhamnosus* R15	Breast milk	MF689049	(*18*)
10	*Lacticaseibacillus rhamnosus* BD2	Kefir granules	MT020089.1	(*18*)
11	*Pediococcus acidilactici* NG64	Cassava tapai	MG928526	(*18*)
12	*Leuconostoc mesenteroides* S2SR08	Tempe	MF164053	(*18*)
13	*Lactobacillus kefiri* JK6	Kefir granules	MT613694.1	(*18*)
14	*Lactobacillus kefiri* BG8	Kefir granules	MT613703.1	(*18*)

### DNA extraction

Each pure culture was grown for 18 h in MRSB supplemented with 10 % glycine. Genomic DNA was extracted from bacterial cell pellets using the Wizard® Genomic DNA Purification Kit (A1120; Promega Corporation, Madison, WI, USA), following the manufacturer's instructions (*27*). The purity and concentration of the extracted DNA were determined using a Nanodrop ND-1000 spectrophotometer (Thermo Fisher Scientific Inc., Waltham, MA, USA). The DNA samples were then stored at −20 °C.

### Primer design

The presence of eight genes involved in the folate biosynthesis pathway was detected in various LAB species by polymerase chain reaction (PCR) amplification. The primers for the detection and sequencing of each gene are shown in Table 2 (*24*). Six of these genes were designed using the online tool NCBI Primer-BLAST (the Basic Local Alignment Search Tool) (*28*) and gene sequences of *Lactiplantibacillus plantarum* WCFS1 obtained from the Kyoto Encyclopedia of Genes and Genomes (KEGG) (*26*) and GenBank (*29*) databases. All primers were manufactured by Integrated DNA Technologies Pte. Ltd. (Coralville, IA, USA).

**Table 2:** List of primers used for the gene detection and sequencing analysis in this study

Targeted gene	Enzyme	Sequence (5′– 3′)	Primer length/base	Amplicon size/bp	*w*(GC)/%	Melting temperature/°C	Reference
*folK*	Hydroxymethyl dihydropteridine pyrophosphokinase (EC 2.7.6.3)	F: CCATTTCCAGGTGGGGAATC	20	214	55.0	55.8	(*24*)
		R: GGGGTGGTCCAAGCAAACTT	20		55.0	58.2	
*folP*	Dihydropteroate synthase (EC 2.5.1.15)	F: CCASGRCSGCTTGCATGAC	19	261	65.8	60.8	(*24*)
		R: TKACGCCGGACTCCTTTTWY	20		50.0	55.8	
*folQ*	Dihydroneopterin triphosphate pyrophosphohydrolase (EC 3.6.1.-)	F: GGCTTGACTGCTCGTCAGTA	20	214	55.0	56.9	*designed in this study
		R: TGACTGCAACCCCTAAGTCG	20		55.0	57.0	
*folE*	GTP cyclohydrolase I (EC 3.5.4.16)	F: CGGGTTGCACGAATGTATGC	20	272	55.0	57.1	*designed in this study
		R: ACTGTCAACCGCTCCTGAAC	20		55.0	57.4	
*folA*	Dihydrofolate reductase (EC 1.5.1.3)	F: GACATGCAGCGGTTCAAAGC	20	362	55.0	57.5	*designed in this study
		R: ACCGTCCCAATTTGTTGGCT	20		50.0	57.7	
*folB*	Dihydroneopterin aldolase (EC 4.1.2.25)	F: GGAAGAACGGCGTAATGGTC	20	263	55.0	56.0	*designed in this study
		R: TTCCAGGCATTGGTACGCTA	20		50.0	56.3	
*folC_1_*	Dihydrofolate synthase (EC 6.3.2.12)	F: AGTGAGCGATTTGGACAGCA	20	331	50.0	57.0	*designed in this study
		R: AGTCGCTGCCATCCTTGAAA	20		50.0	57.1	
*folC_2_*	Folylpolyglutamate synthase (EC 6.3.2.17)	F: GGCTGTTTTGCAGACCGAAG	20	487	55.0	57.0	*designed in this study
		R: TGCGGGCGTATTCGTAATCA	20		50.0	56.7	

GC=guanine-cytosine

### Polymerase chain reaction amplification for the gene detection

PCR amplifications were performed in 20 µL volumes with 10 µL Promega Go Taq Green Master Mix 1× (M7122; Promega Corporation), containing Taq DNA polymerase, dNTPs, MgCl_2_ and reaction buffers, 1 µL of 0.5 µM of each forward and reverse primer, 1 µL DNA template (>150 ng) and 7 µL nuclease-free water (NFW P1193; Promega Corporation). The amplifications were carried out using an Applied Biosystems 2720 Thermal Cycler (Applied Biosystems, Foster City, CA, USA) with the following cycling conditions: 1 cycle at 95 °C for 2 min; 30–35 cycles at 95 °C for 30 s, the annealing temperature depending on the melting temperature of each primer and tested at 50–60 °C for 1 min, and at 72 °C for 30 s; and 1 cycle at 72 °C for 5 min. The positive and negative controls were also subjected to amplification. The positive control was DNA from the reference strain (*L. plantarum* WCFS1), while the negative control was NFW without DNA containing the target genes. The PCR products were then separated by gel electrophoresis (Mini-Sub Cell GT Horizontal Electrophoresis System; BioRad Laboratories Inc., Hercules, CA, USA) using 2 % agarose gel in 1× Tris-acetate-EDTA (TAE) buffer and visualized using ethidium bromide staining.

### Sequencing analysis

The detected folate biosynthetic genes in six folate-producing isolates of different species, represented by *Lactiplantibacillus plantarum* 4C261, *Lacticaseibacillus rhamnosus* R23, *Limosilactobacillus fermentum* JK13 and BG7, *Pediococcus acidilactici* NG64, and *Leuconostoc mesenteroides* S2SR08, were sequenced by 1st Base Sequencing (Selangor, Malaysia) using the Sanger method (Sanger dideoxy sequencing) (*30*) after purification. A single sample of purified DNA for each folate gene was needed as a template for sequencing. The PCR products with more than one band on agarose gel electrophoresis were first cut out of the gel and purified using a DNA gel extraction kit (GeneJET Gel Extraction Kit; Thermo Fisher Scientific Inc.). Samples with a single DNA band of the expected size on agarose gel electrophoresis were purified further before sequencing. The PCR cleanup step was then performed by an ultrafiltration method using Centricon-100 micro-concentrator columns (Applied Biosystems) to remove unincorporated primers and dNTPs that can interfere with the sequencing results. DNA quality and quantity were then determined by agarose gel electrophoresis and spectrophotometry. After DNA template preparation, cycle sequencing was performed using the BigDye Terminator v3.1 cycle sequencing kit (Applied Biosystems) and run in a thermal cycler (GeneAmp PCR System 9700; Applied Biosystems). The excess dye terminator was removed by spin column purification with Centri-Sep spin columns (Applied Biosystems) prior to analysis on an ABI PRISM 3730xl genetic analyzer (Applied Biosystems). The primers used for sequencing were those listed in Table 2 (the same primers for gene detection). The sequencing output was then analyzed using Sequence Scanner Software v. 2.0 (Applied Biosystems) (*31*).

The sequenced nucleotides were processed using MEGA7: Molecular Evolutionary Genetic Analysis v. 7.0 (*32*). The obtained consensus DNA sequences (contigs) were used as the query sequences to perform homology searches using the NCBI BLAST algorithm (*28*). The 'blastn' program was used to find areas of local similarity between query and database nucleotides. The identified query sequences were then stored in the GenBank database with the accession numbers shown in Table 3 (*29*).

**Table 3:** GenBank (*29*) accession numbers of each gene

Gene	GenBank accession no.					
4C261	R23	JK13	BG7	S2SR08	NG64
*folE*	OP067669	OP067670	OP067671	OP067672	OP067673	OP067674
*folQ*	ON972433	ON986778	ON972435	ON972434	ON986777	ON972436
*folB*	OP032089	OP032086	OP032085	OP032088	OP032084	OP032087
*folK*	OP032090	OP032091	OP032092	OP032093	OP032094	OP032095
*folP*	OP067663	OP067664	OP067665	OP067666	OP067667	OP067668
*folC_1_*	OP081807	OP081808	OP081809	OP081810	OP081811	OP081812
*folA*	ON950739	ON972429	ON972430	ON972428	ON972432	ON972431
*folC_2_*	OP067675	OP067676	OP067677	OP067678	OP067679	OP081806

### Phylogenetic tree construction

Due to the inadequacy of the folate biosynthesis gene database in NCBI GenBank (*29*), the KEGG (*26*) was used as a primary reference for nucleotide sequence database for each folate biosynthetic gene. The MEGA v. 7.0 software (*32*) was used to align folate-related gene sequences from six isolates and reference data by the ClustalW program (incorporated in the MEGA software). The same software was also used to create phylogenetic trees using the neighbor-joining method and Jukes and Cantor models for determining evolutionary distance values with a 1000 replicate bootstrap test.

### Extracellular folate production in folate-free medium

The inoculum of all LAB isolates was prepared according to a previously described method (*18*). Bacterial cell pellets grown for 24 h in MRSB were washed twice by centrifugation (refrigerated centrifuge LRF-B20; Labtron Equipment Ltd., Camberley, UK) at 10 000×*g* for 5 min at 4 °C and finally resuspended in sterile saline solution (0.85 % *m*/*V* NaCl; Merck KGaA, Darmstadt, Germany). The bacterial suspensions had cell densities of around 9–10 log colony-forming units (CFU)/mL, which were diluted to 5–6 log CFU/mL before use as inoculum. A total of 2 % inoculum was then grown in folate-free medium (folic acid casei medium, FACM; HiMedia Laboratories, Mumbai, India) at 37 °C for 24 h and subcultured twice in the same medium under the same growth conditions. The extracellular folate was extracted by centrifugation (refrigerated centrifuge LRF-B20; Labtron Equipment Ltd.) at 10 000×*g* for 5 min, followed by supernatant filtration using a 0.2-μm nylon filter membrane (ANPEL Laboratory Technologies Inc., Shanghai, PR China). All samples were then stored at –20 °C until further analysis.

### Extracellular folate analysis with HPLC

Extracellular folate was analyzed using an Agilent 1260 Infinity HPLC System with a diode array detector (DAD; Agilent Technologies Inc., Santa Clara, CA, USA) and a ZORBAX Eclipse XDB-C18 chromatography column (15 cm×4.6 mm, 5 μm; Agilent Technologies, Inc.) at *λ*=282 nm. The mobile phase was freshly prepared and consisted of water (HPLC grade; LiChrosolv®, Merck KGaA) with glacial acetic acid (0.66 %; EMSURE®, Merck KGaA) and methanol (pure HPLC grade; LiChrosolv®, Merck KGaA), with a ratio of *V*(water):*V*(methanol)=70:30 (*33*). The flow rate was 0.8 mL/min. Folic acid standard was obtained from R-Biopharm (provided in the Vitafast folic acid test kit; Pfungstadt, Germany) and used without further purification.

### Data analysis

The data were analyzed using SPSS v. 20.0 (*34*), with the statistical significance level set at 95 % (α<0.05). A one-way ANOVA was used to compare the differences in the extracellular folate productivity of the isolates in folate-free culture medium (FACM).

## RESULTS AND DISCUSSION

### Detection of folate biosynthesis genes

**Fig. 1:**
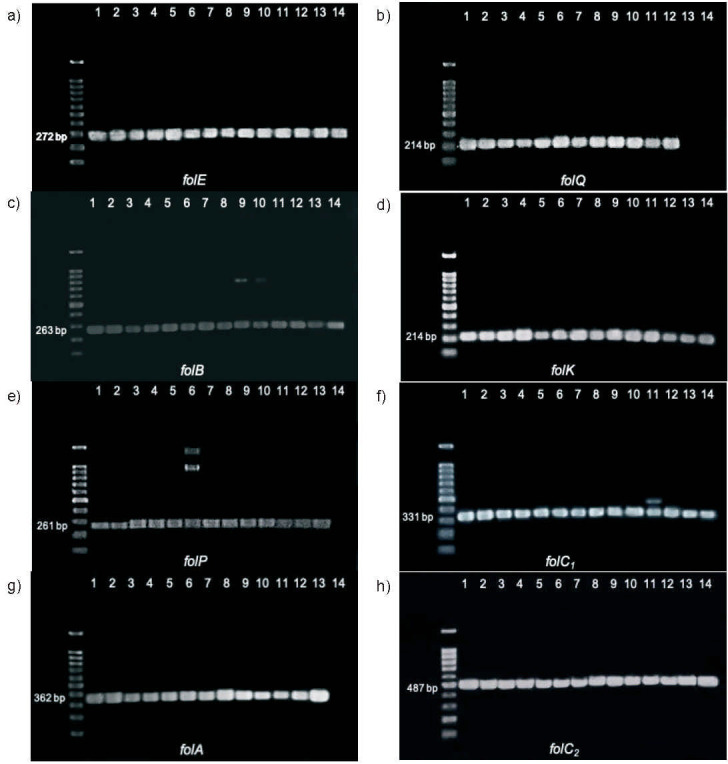
Detection of folate biosynthetic genes: a) *folE*, b) *folQ*, c) *folB*, d) *folK*, e) *folP*, f) *folC_1_*, g) *folA*, and h) *folC_2_*, by gel electrophoresis in 14 isolates of lactic acid bacteria. Lane 1: *Lactiplantibacillus plantarum* WCFS1, lane 2: *L. plantarum* 4C261, lane 3: *L. plantarum* R12, lane 4: *Lacticaseibacillus rhamnosus* R23, lane 5: *L. rhamnosus* R15, lane 6: *L. rhamnosus* BD2, lane 7: *Limosilactobacillus fermentum* JK13, lane 8: *L. fermentum* JK16, lane 9: *L. fermentum* BK27, lane 10: *L. fermentum* BG7, lane 11: *Leuconostoc mesenteroides* S2SR08, lane 12: *Pediococcus acidilactici* NG64, lane 13: *Lactobacillus kefiri* JK6, and lane 14: *L. kefiri* BG8

Eight folate biosynthetic genes (*folE*, *folQ*, *folB* and *folK* in the DHPPP formation pathway, and *folP*, *folC_1_*, *folA* and *folC_2_* in the THF-polyglutamate formation pathway) were successfully amplified using gene-specific primers, with the predicted size of the PCR amplicon for each gene (Fig. 1). As a positive control, a single band for each of the eight genes was also detected in *Lactobacillus plantarum* WCFS1. No bands were detected in the negative control (data not shown).

However, despite designing specific primers and performing amplifications at various annealing temperatures, non-specific detections, seen as a double band or multiple bands on gel electrophoresis, were observed. In seven of the 11 folate-producing isolates, *i.e. Lactobacillus rhamnosus* R23 and R15, *L. fermentum* JK13, *L. plantarum* 4C261, R12 and JK16, and *Pediococcus acidilactici* NG64 (*18*), all folate biosynthetic genes were specifically detected. In the other folate-producing isolates, there were non-specific detections of one gene, *i.e*. *L. fermentum* BK27 (*folB*), *L. fermentum* BG7 (*folB*), *Leuconostoc mesenteroides* S2SR08 (*folC_1_*) and *L. rhamnosus* BD2 (*folP*). Non-specific detection of genes was previously reported by Saubade *et al.* (*35*), where the *folP* gene was detected in several pearl-millet-based porridge samples by more than one band on gel electrophoresis. The report also suggested that both specific and non-specific detections were thought to indicate the presence of a gene.

Two isolates, JK6 and BG8, are known folate non-producers (*18*) and did not have a complete set of folate biosynthesis genes, possibly leading to their inability to produce folate. The JK6 isolate lacked the *folQ* gene, while the BG8 isolate lacked *folQ, folP* and *folA*, as indicated by the absence of the corresponding bands after gel electrophoresis (Fig. 1b, Fig. 1e and Fig. 1g). However, the two non-folate-producing isolates still have *folK*, one of the signature genes for folate biosynthesis, and even the JK6 isolate has both signature genes, *folK* and *folP*. This finding is supported by Greppi *et al*. (*9*), who reported that some LAB isolates could not synthesize folate despite having the signature genes *folP* and *folK*. Other folate biosynthetic genes probably play an essential role in determining the LAB folate production capacity.

Tetrahydrofolate, whose production is regulated by *folP*, *folC_1_*, *folA* and *folC_2_*, acts as a cofactor in one-carbon metabolic reactions in various pathways, such as the biosynthesis of purines, thymidine, glycine, methionine, pantothenate and formyl-methionyl tRNA (fMet-tRNA), which is required for the initiation of protein synthesis (*23*, *36*–*39*). These folate-dependent metabolites, except fMet-tRNA, can be provided in bacterial growth media. Thus, THF is essential for the production of fMet-tRNA, which is needed for the growth of folate-requiring bacteria. In the final stage of the THF biosynthetic pathway, the reduction of dihydrofolate to THF is catalyzed by dihydrofolate reductase (DHFR), encoded by the *folA* gene. In addition, when bacteria require a folate supply from the environment, the conversion of folic acid to dihydrofolate in the folate salvage pathway also involves the activity of the DHFR enzyme (*23*). For this reason, all folate-dependent bacteria need a minimum amount of DHFR activity to synthesize fMet-tRNA. Thus, DHFR is expected to be found in both folate producers and non-producers (*23*, *40*).

However, not all LAB isolates in our investigation had the *folA* gene. The BG8 isolate, a non-producer of folate, lacked this gene. Levin *et al*. (*41*) also stated that although THF is an essential cofactor for all bacteria, the gene encoding DHFR cannot be found in many bacteria. The unavailability of the *folA* gene in these bacteria may be due to the presence of another type of DHFR enzyme encoded by another gene. Work by de Crécy-Lagard *et al*. (*23*) showed that some bacteria might have different types of DHFR, such as DHFR1 (encoded by *folM*), belonging to the short-chain dehydrogenase/reductase family, or DHFR2, a flavin-dependent dihydropteroate reductase fused with DHFR. Hence, the BG8 isolate is predicted to also have the *folM* gene or the gene encoding DHFR2, as a homologous gene of *folA*.

In the folate biosynthetic pathway, the formation of THF-polyglutamate is crucial for folate retention in bacterial cells and is required for most folate-dependent enzymes which have a higher affinity for polyglutamate folate (*6*, *42*, *43*). The conversion of THF monoglutamate into the polyglutamate form is catalyzed by folylpolyglutamate synthase (FPGS), encoded by the *folC_2_* gene. Therefore, in addition to *folA*, *folC_2_* should also be found in all folate-dependent bacteria. Furthermore, FPGS is essential for folate-consuming bacteria, as they can only sequester monoglutamate folate (1-3 glutamate residues) *via* the salvage route due to the lack of an enzyme to break down folate polyglutamate (γ-glutamyl hydrolase) (*20*, *44*, *45*). However, in some bacteria, the *folC_2_* gene can be found in the fusion gene of *folC*, encoding the bifunctional enzymes DHFS and FPGS. Non-folate-producing bacteria lacking either the *folC_2_* or *folC* genes may harbour a gene encoding a novel type of FPGS. According to de Crecy-Lagard *et al*. (*23*), the unavailability of the *folC* gene in *Mycoplasma* species relying on the salvage pathway may be due to the presence of another gene encoding a different FPGS. In this study, *folC_2_* was detected in all LAB isolates (producers and non-producers of folate).

Since all folate-dependent bacteria have the genes *folA* and *folC_2_* (or *folC*), or their homologues (*23*, *40*), folate biosynthetic genes defining the potential ability of LAB to synthesize folate may include genes *folE*, *folQ*, *folB* and *folK*, involved in the DHPPP biosynthetic pathway, and the *folP* gene, involved in THF-polyglutamate biosynthesis. Rossi *et al*. (*12*) also reported that the genes *folC* (*folC_1_*/*folC_2_*) and *folA*, or their homologues, were found in all studied LAB species, whereas other folate biosynthetic genes were detected only in a few species.

In this study, the JK6 and BG8 isolates lacked the gene *folQ*, which encodes dihydroneopterin triphosphate pyrophosphohydrolase. In some folate-producing bacteria (such as *Bacillus subtilis*, *Escherichia coli* and *Streptococcus thermophilus*), the absence of the *folQ* gene may be due to the presence of other homologous genes that have not been identified (*25*). However, as JK6 and BG8 isolates could not grow in a folate-free medium (FACM), the missing *folQ* gene in both isolates may indicate that the gene *folQ* can be a limiting factor in their ability to synthesize folate. Moreover, Liu *et al*. (*46*) have reported that in the DHPPP biosynthetic pathway, the *folQ* gene had the highest expression level, demonstrating a better folate synthesis ability in *L. plantarum* strain 4_3 used in their study. Although further research of gene expression level analysis is also required in this study, the present findings suggest that the *folQ* gene may play a crucial role in determining the potential ability of LAB to synthesize folate.

Here, the detection of folate biosynthetic genes did not include the detection of genes encoding PABA biosynthetic enzymes (*pabA* and *pabB*/*pabC*) due to the lack of these genes in almost all lactobacilli, as reported by Rossi *et al*. (*12*). Thus, lactobacilli are generally unable to produce folate without PABA supplementation in the medium (*9*, *12*). The FACM used in this study has neither folate nor glutamic acid but it still contains 2 mg/L of PABA (based on the technical datasheet). Despite the presence of PABA in the FACM, the JK6 and BG8 isolates were unable to grow in this medium, indicating that both could not utilize PABA as a precursor in the medium to carry out folate biosynthesis in their cells. Hence, the availability of PABA biosynthetic genes can be neglected in this case.

### Phylogenetic analyses

**Fig. 2:**
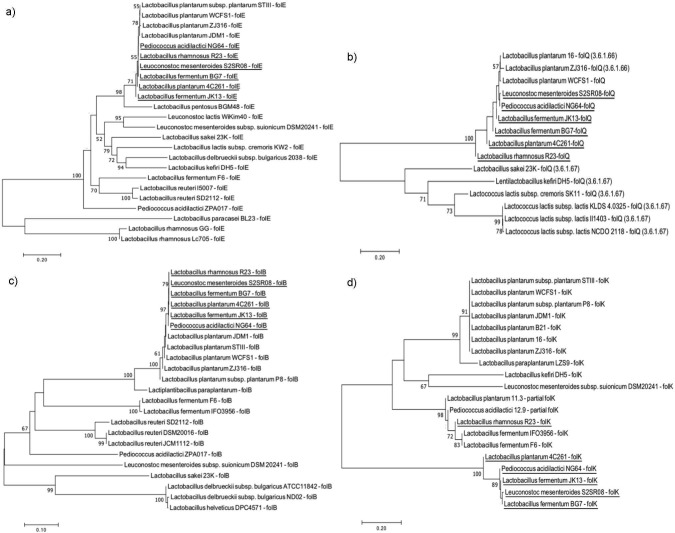
Phylogenetic analyses of: a) *folE*, b) *folQ*, c) *folB*, and d) *folK* genes involved in the 6-hydroxymethyl-7,8-dihydropterin pyrophosphate (DHPPP) biosynthesis pathway by 6 folate-producing lactic acid bacteria (LAB). The phylogenetic trees were constructed using the Jukes and Cantor model and the neighbor-joining method included in MEGA v. 7 software (*32*). Bootstrap values (based on 1000 replicates) that are greater than 50 % are indicated at the nodes

**Fig. 3:**
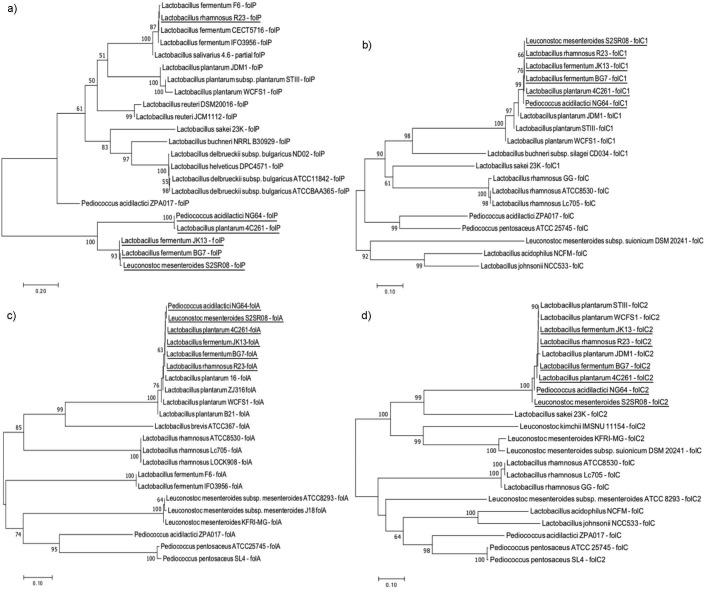
Phylogenetic analyses of: a) *folP*, b) *folC_1_*, c) *folA*, and d) *folC_2_* genes involved in the tetrahydrofolate (THF)-polyglutamate biosynthesis pathway by 6 folate-producing lactic acid bacteria (LAB). The phylogenetic trees were constructed using the Jukes and Cantor model and the neighbor-joining method included in MEGA v. 7 software (*32*). Bootstrap values (based on 1000 replicates) that are greater than 50 % are indicated at the nodes

The sequences of eight folate biosynthetic genes (*folE*, *folQ*, *folB*, *folK*, *folP*, *folC_1_*, *folA* and *folC_2_*) from six folate-producing LAB isolates (R23, 4C261, JK13, BG7, S2SR08 and NG64) (using the same primer for gene detection) yielded different contig sizes for each isolate (Table S1). Nucleotide BLAST search revealed that all the folate genes are homologous with the LAB genome (instead of specific genes for folate biosynthesis) in the NCBI database with 100 % nucleotide sequence identity (Table S2). Hence, the references of nucleotide sequences for each folate biosynthetic gene from KEGG database were used to construct the phylogenetic trees shown in Fig. 2 and Fig. 3. The phylogenetic tree analyses showed that the six folate genes (*folE*, *folQ*, *folB*, *folA*, *folC_1_* and *folC_2_*) found in folate-producing isolates are from the monophyletic group of LAB species in the database, including the positive control, *L. plantarum* WCFS1 (Figs. 2a-2c and Figs. 3b-3d). For the two other genes, *folK* and *folP,* only isolate R23 has both genes from the monophyletic group of LAB species in the database, while the other five folate-producing isolates have the genes from the polyphyletic group (Fig. 2d and Fig. 3a).

*L. plantarum* WCFS1 was selected as a reference strain to compare the similarity of the folate genes from the six isolates because it possesses a complete set of folate biosynthetic genes and has been well studied for its capacity to produce folate (*9*, *24*–*26*). The comparison of the number of conserved sites (C) and variable sites (V) of eight folate biosynthetic genes among the six folate-producing LAB isolates and the reference strain (*L. plantarum* WCFS1) is given in Table S3. A higher percentage of conserved sites was found in six folate genes that are monophyletic with *L. plantarum* WCFS1, ranging from 66 to 86 % for *folE,* 47–96 % for *folQ*, 96–98 % for *folB*, 95 % for *folC_1_*, 66–97 % for *folA*, and 92–96 % for *folC_2_*. Only 0–2 % of sites were variable sites and the rest were gaps. Meanwhile, the two folate genes (*folK* and *folP*) of five isolates that are polyphyletic with *L. plantarum* WCFS1 showed fewer conserved sites (21–30 % for *folK* and 20–23 % for *folP*) than the variable sites (33–53 % for *folK* and 39–54 % for *folP*). As an exception, R23 had a higher proportion of conserved sites for both genes, as it is monophyletic with WCFS1.

Six folate-producing LAB isolates have the genetic capacity for *de novo* folate biosynthesis. The R23 isolate had all homologous folate genes that were the same as the positive control, *L. plantarum* WCFS1. The nucleotide sequences of the *folK* and *folP* genes of the five isolates showed remarkable differences from those of the positive control. The higher variable sites of *folK* and *folP* in five LAB isolates may indicate that both genes are not essential in the folate biosynthesis pathway. Jordan *et al.* (*47*) stated that essential genes are more conserved than nonessential genes in bacteria. The *folK* and *folP* genes of five isolates may contribute at different levels to *de novo* folate biosynthesis. However, more research is required to determine their correlation with folate production ability. The selection of *folQ* as a marker gene for folate biosynthesis in this study may also indicate that the *folQ* gene is essential in the folate biosynthesis pathway.

Based on the phylogenetic tree in Fig. 2b, the gene *folQ*, which encodes the enzyme that catalyzes the conversion of dihydroneopterin triphosphate to dihydroneopterin monophosphate, is known to produce two isoforms, XTP/dITP diphosphatase (EC 3.6.1.66) and dihydroneopterin triphosphate diphosphatase (EC 3.6.1.67), that have the same function. Several LAB species from the KEGG database, such as *Latilactobacillus sakei*, *L. kefiri* and *Lactococcus lactis,* have the *folQ* gene encoding dihydroneopterin triphosphate diphosphatase (EC 3.6.1.67), while *L. plantarum* species has the *folQ* gene, which encodes for the XTP/dITP diphosphatase. These two types of enzymes were divided into two different clades (Fig. 2b). The *folQ* genes of six folate-producing LAB isolates (R23, 4C261, JK13, BG7, S2SR08 and NG64) in this study were in the same cluster as *L. plantarum*, indicating that the enzyme encoded by the *folQ* gene of the six isolates was probably the XTP/dITP diphosphatase.

### Extracellular folate production

**Fig. 4:**
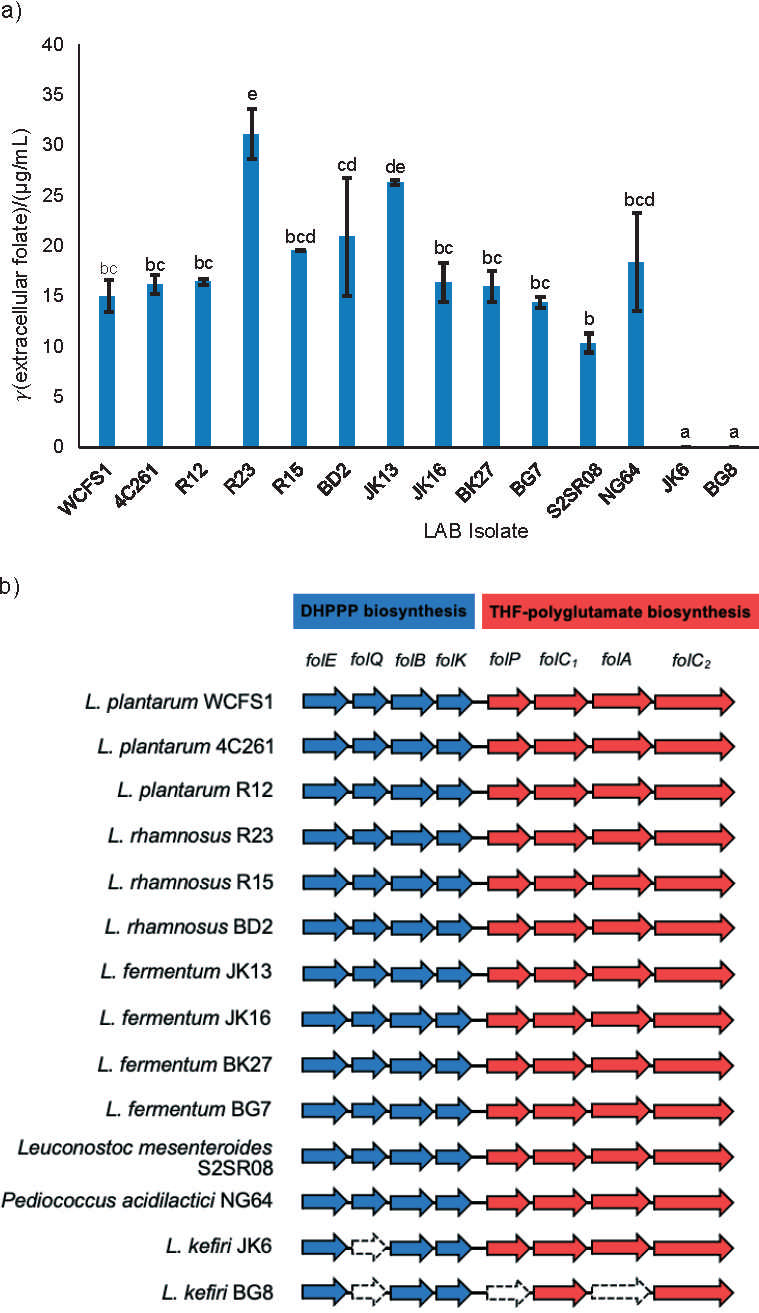
Comparison of extracellular folate productivity and folate biosynthesis gene availability in 14 isolates of lactic acid bacteria (LAB). a) Extracellular folate productivity of 14 LAB isolates in folic acid casei medium (FACM) after 24 h of growth. The different letters (a-e) above the bars indicate significant differences between the means (p<0.05). b) Presence of folate biosynthetic genes in 14 LAB isolates. Arrow length represents gene length, on a scale of 0.1 cm=40 bp. DHPPP=6-hydroxymethyl-7,8-dihydropterin pyrophosphate, THF=tetrahydrofolate

The concentration of folate produced by eleven LAB isolates (4C261, R12, R23, R15, BD2, JK13, JK16, BK27, BG7, S2SR08 and NG64) ranged from 10.37 to 31.10 μg/mL (Fig. 4a). The variability in the concentration of folate produced by these isolates was related to the availability of all eight genes involved in synthesizing folate (Fig. 4b). However, two isolates, JK6 and BG8, did not secrete extracellular folate (Fig. 4a) and did not have all the genes for folate biosynthesis (Fig. 4b). Five isolates (R23, JK13, BD2, R15 and NG64) had higher extracellular folate productivity, ranging from 18.40 to 31.10 μg/mL, than that of the positive control, *L. plantarum* WCFS1. The isolate *L. rhamnosus* R23 was the highest folate producer, producing 207 % more folate than WCFS1.

The folate concentrations measured in the isolates in this study were higher than those reported by Kodi *et al.* (*33*), who studied the LCF10 LAB strain isolated from fermented milk. Using the same analytical method employed here, they found that LCF10 produced 10.4 μg/mL folate in FACM after 18 h of incubation. Wu *et al*. (*48*) found higher folate production concentrations in *L. casei*, *L. acidophilus* and *L. plantarum*, which produced 45.41, 42.78 and 63.23 μg/mL of folate in yogurt products, respectively. Nevertheless, the highest folate concentration (31.10 μg/mL) produced by isolate R23 in this study is equivalent to ~8 % of the recommended daily folate intake, which is 400 μg per day for the average adult (*49*). Supplementation of food products with the R23 isolate can potentially be used as a good source of natural folate.

## CONCLUSIONS

All eight folate biosynthetic genes were detected in eleven folate-producing LAB isolates. In the two non-folate-producing isolates, not all folate biosynthetic genes were present. The majority of the identified genes were homologous to genes encoding enzymes involved in the folate biosynthetic pathway, confirming the necessity of these genes in the LAB for folate production ability. The present study also suggests that molecular detection and identification is an excellent strategy for screening folate-producing LAB as an alternative to phenotypic analysis, which is quite time-consuming, laborious and costly. The *folQ* gene, which was not detected in non-folate-producing isolates, could potentially be used as a marker for folate biosynthesis.

## Appendix

**Table S1:** Contig size of folate biosynthetic genes from six folate-producing lactic acid bacteria (LAB) isolates

Isolate	*folE*	*folQ*	*folB*	*folK*	*folP*	*folC_1_*	*folA*	*folC_2_*
R23	273	123	265	131	163	333	273	478
4C261	271	128	265	142	194	332	364	470
JK13	209	115	257	215	185	333	248	478
BG7	274	124	265	206	185	333	260	487
S2SR08	274	216	265	208	194	333	364	488
NG64	273	105	257	209	233	331	272	487

**Table S2:** Percent identity of folate biosynthetic genes from six folate-producing lactic acid bacteria (LAB) with nucleotide sequences from various LAB species in the NCBI databases (*29*)

Gene	Isolate	NCBI blastn	
Identity/ %	Description
*folE*	4C261	100	*L. plantarum* strain SKO-001 chromosome, complete genome; ID: CP040374.1
	R23	100	*L. plantarum* strain TCI507 chromosome, complete genome; ID: CP054259.1
	JK13	100	*L. plantarum* strain Heal19 chromosome, complete genome; ID: CP055123.1
	BG7	100	*L. plantarum* strain TCI507 chromosome, complete genome; ID: CP054259.1
	NG64	100	*L. plantarum* strain TCI507 chromosome, complete genome; ID: CP054259.1
	S2SR08	100	*L. plantarum* strain TCI507 chromosome, complete genome; ID: CP054259.1
*folQ*	4C261	100	*L. plantarum* strain SRCM102737 chromosome, complete genome; ID: CP028261.1
	R23	100	*L. plantarum* strain Heal19 chromosome, complete genome; ID: CP055123.1
	JK13	100	*L. plantarum* strain Heal19 chromosome, complete genome; ID: CP055123.1
	BG7	100	*L. plantarum* strain Heal19 chromosome, complete genome; ID: CP055123.1
	NG64	100	*L. plantarum* strain SRCM103418 chromosome, complete genome; ID: CP035168.1
	S2SR08	100	*L. plantarum* strain SRCM103418 chromosome, complete genome; ID: CP035168.1
*folB*	4C261	100	*L. plantarum* strain Heal19 chromosome, complete genome; ID: CP055123.1
	R23	100	*L. plantarum* strain Heal19 chromosome, complete genome; ID: CP055123.1
	JK13	100	*L. plantarum* strain Heal19 chromosome, complete genome; ID: CP055123.1
	BG7	100	*L. plantarum* strain Heal19 chromosome, complete genome; ID: CP055123.1
	NG64	100	*L. plantarum* strain Heal19 chromosome, complete genome; ID: CP055123.1
	S2SR08	100	*L. plantarum* strain Heal19 chromosome, complete genome; ID: CP055123.1
*folK*	4C261	100	*L. fermentum* strain LfQi6 chromosome; ID: CP025592.1
	R23	100	*L. fermentum* strain LfQi6 chromosome; ID: CP025592.1
	JK13	100	*L. fermentum* strain LfQi6 chromosome; ID: CP025592.1
	BG7	100	*L. fermentum* strain LfQi6 chromosome; ID: CP025592.1
	NG64	100	*L. fermentum* strain LfQi6 chromosome; ID: CP025592.1
	S2SR08	100	*L. fermentum* strain LfQi6 chromosome; ID: CP025592.1
*folP*	4C261	100	*L. plantarum* strain Heal19 chromosome, complete genome; ID: CP055123.1
	R23	100	*L. fermentum* strain HFD1 chromosome, complete genome; ID: CP050919.1
	JK13	100	*L. fermentum* strain HFD1 chromosome, complete genome; ID: CP050919.1
	BG7	100	*L. fermentum* strain HFD1 chromosome, complete genome; ID: CP050919.1
	NG64	100	*L. plantarum* strain Heal19 chromosome, complete genome; ID: CP055123.1
	S2SR08	100	*L. fermentum* strain HFD1 chromosome, complete genome; ID: CP050919.1
*folC_1_*	4C261	100	*L. plantarum* strain Heal19 chromosome, complete genome; ID: CP055123.1
	R23	100	*L. plantarum* strain Heal19 chromosome, complete genome; ID: CP055123.1
	JK13	100	*L. plantarum* strain Heal19 chromosome, complete genome; ID: CP055123.1
	BG7	100	*L. plantarum* strain Heal19 chromosome, complete genome; ID: CP055123.1
	NG64	100	*L. plantarum* strain Heal19 chromosome, complete genome; ID: CP055123.1
	S2SR08	100	*L. plantarum* strain Heal19 chromosome, complete genome; ID: CP055123.1
*folA*	4C261	100	*L. plantarum* strain TCI507 chromosome, complete genome; ID: CP054259.1
	R23	100	*L. plantarum* strain TCI507 chromosome, complete genome; ID: CP054259.1
	JK13	100	*L. plantarum* strain TCI507 chromosome, complete genome; ID: CP054259.1
	BG7	100	*L. plantarum* strain TCI507 chromosome, complete genome; ID: CP054259.1
	NG64	100	*L. plantarum* strain Heal19 chromosome, complete genome; ID: CP055123.1
	S2SR08	100	*L. plantarum* strain NCIMB8826 (WCFS1) chromosome, complete genome; ID: CP037961.1
*folC_2_*	4C261	100	*L. plantarum* strain LLY-606 chromosome, complete genome; ID: CP023306.1
	R23	100	*L. plantarum* strain Heal19 chromosome, complete genome; ID: CP055123.1
	JK13	100	*L. plantarum* strain Heal19 chromosome, complete genome; ID: CP055123.1
	BG7	100	*L. plantarum* strain Heal19 chromosome, complete genome; ID: CP055123.1
	NG64	100	*L. plantarum* strain SRCM100442 chromosome, complete genome; ID: CP028221.1
	S2SR08	100	*L. plantarum* strain SRCM100442 chromosome, complete genome; ID: CP028221.1

**Table S3:** The comparison of the number of conserved sites (C) and variable sites (V) of eight folate biosynthetic genes between six folate-producing lactic acid bacteria (LAB) isolates and the reference strain (*Lactiplantibacillus plantarum* WCFS1)

Isolate	Reference strain (WCFS1)															
*folE*		*folQ*		*folB*		*folK*		*folP*		*folC_1_*		*folA*		*folC_2_*	
C	V	C	V	C	V	C	V	C	V	C	V	C	V	C	V
R23	271/314	2/314	123/221	0/221	263/268	2/268	72/257	59/257	92/302	68/302	327/345	6/345	272/374	1/374	478/509	0/509
4C261	269/314	2/314	127/221	1/221	263/268	2/268	53/257	84/257	59/302	134/302	327/345	5/345	361/374	3/374	469/509	1/509
JK13	209/314	0/314	115/221	0/221	256/268	1/268	76/257	134/257	65/302	119/302	327/345	6/345	247/374	1/374	478/509	0/509
BG7	271/314	3/314	124/221	0/221	263/268	2/268	73/257	128/257	65/302	119/302	327/345	6/345	259/374	1/374	487/509	0/509
S2SR08	271/314	3/314	212/221	4/221	263/268	2/268	73/257	130/257	67/302	126/302	327/345	6/345	362/374	2/374	484/509	4/509
NG64	271/314	2/314	104/221	1/221	256/268	1/268	74/257	130/257	70/302	162/302	327/345	4/345	272/374	0/374	483/509	4/509
